# Impact of Nursing Education on CLABSI Rates: An Experience from a Tertiary Care Hospital in Eastern India

**DOI:** 10.5005/jp-journals-10071-23205

**Published:** 2019-07

**Authors:** Ranjita Acharya, Shakti Bedanta Mishra, Snigdha Ipsita, Afzal Azim

**Affiliations:** 1 Department of Anesthesiology, IMS and SUM Hospital, Bhubaneswar, Odisha, India; 2,3 Department of Critical Care Medicine, IMS and SUM Hospital, Bhubaneswar, Odisha, India; 4 Department of Critical Care Medicine, SGPGIMS, Lucknow, Uttar Pradesh, India

**Keywords:** Central line associated bloodstream infections, hand hygiene

## Abstract

**Background and Aims:**

Central line associated bloodstream infections (CLABSI) have a higher incidence in the intensive care units of developing countries.

**Materials and Methods:**

The baseline CLABSI rate in intensive care unit (ICU) was evaluated for 6 months. An educational program for nurses on basic hand hygiene steps was conducted. Objective assessment tests were done to assess their knowledge and percentage of non-compliance with hand hygiene practice. CLABSI rate over the post-intervention 6 month period was assessed.

**Results:**

Thirty-four nurses were enrolled. The pre-intervention CLABSI rate was 12.5 per 1000 catheter days, pretest score 15.9 +/− 3.35 and 53.4% opportunities for hand hygiene were missed. Post workshop, there was significant (p=0.02) decrease in CLABSI rate i.e. 8.6, improvement in test score 17.76 +/− 2.1 (*p*=0.011) and missed opportunities decreased to 33.75%. 6 months post intervention, percentage of noncompliance with hand hygiene practice were 51.75% and test score was 17 ± 2.

**Discussion:**

The effectiveness of educational program on hand hygiene compliance was reflected in the improvement of posttest score, reduced number of missed opportunities and reduction of CLABSI rates in ICU. The posttest scores and hand hygiene compliance, however, decreased 6 months post-intervention necessitating repeated feedbacks and reminders.

**Conclusion:**

Educational interventions on hand hygiene can have a significant impact in CLABSI control particularly in ICUs with a high infection rate and resource constraints.

**How to cite this article:**

Acharya Ranjita, Mishra SB, Ipsita S, Azim A. Impact of Nursing Education on CLABSI Rates: An Experience from a Tertiary Care Hospital in Eastern India. Indian J Crit Care Med 2019;23(7):316–319.

## INTRODUCTION

Central line associated blood stream infection (CLABSI), one of the most common hospital acquired infections, is a major contributor to in-hospital morbidity and mortality and is associated with increased expenditure and length of intensive care unit (ICU) stay.^1^ The incidence of CLABSI is more in ICUs due to emergency catheter placement, longer duration and repeated manipulation for sampling, administration of drugs and fluids; the additional confounding factors being chronic illness, old age, sepsis and immunosuppresion. In the United States, mean ICU and in patient ward CLABSI rates are 1.6 and 1.1 per 1000 catheter days, respectively^2^ and are close to the target of zero catheter related infections. Though less published data on developing countries is available, the pooled CLABSI rate ranges from 1.6 to 44.6 per 1000 catheter days in adult and paediatric ICUs.^3^

Reduction of catheter related infections was one of the 7 healthcare safety challenges issued by Centers for Disease Control and Prevention.^4^ Hand hygiene is the most convenient and cost effective measure to reduce hospital acquired infections.^5^ Despite this, hand hygiene compliance has less research attention as compared to other strategies.^6^ The compliance to hand hygiene can be promoted by a multidisciplinary approach of education, training, direct observation, performance feedbacks and verbal reminders. Therefore, in an effort to mitigate the CLABSI rates in our ICU, promote performance improvement among the nursing staff, we conducted an education based program on hand hygiene with a before after evaluation to assess the effect of the educational intervention.

## MATERIALS AND METHODS

A quasi-experimental study with regard to education of nursing staff to reduce the incidence of central line associated bloodstream infections (CLABSI) was conducted in medicine ICU of a tertiary care hospital in Eastern India. The study was conducted in three time phases. An educational intervention involving nurses was designed to reduce the catheter related infection rates and a before-after evaluation was carried out to assess the benefits. A pretest and post test design was adopted to analyse how well the nurses are versed with the infection prevention and control practices of central catheter care. CLABSI was defined as a laboratory confirmed bloodstream infection in a patient where the central line was in place for >48 hours with no other apparent source except the catheter. If the central line was present for >48 hours and removed, either of the following two criteria were considered to be termed as CLABSI i.e. one or more positive blood culture with no other apparent source or patient had clinical signs of infection like fever >38°C, chills or hypotension with organism unrelated to another infection site and the same organism cultured from two or more blood samples drawn on separate occasions. The timeframe taken between any two elements like fever and positive blood cultures was 24 hours.^7^ One catheter day was defined as a day on which the patient had one or more catheters. The CLABSI rate was the number of catheter related infections per 1000 catheter days. ^8^

In phase I, the baseline data collection pertaining to the CLABSI rates and compliance to hand hygiene was done over a period of 6 months from November to April 2016.A pre-intervention test was conducted to assess the central venous catheter care practices among the nurses. In phase II, an educational intervention was provided highlighting the procedures and practices for the prevention and control of CLABSI. The workshop comprised of a 30 minutes didactic lecture and 30 minutes bedside demonstration on hand hygiene and basic clinical practices for reduction of central venous catheter infections. An objective test was conducted among the nurses after the workshop. Phase III was the period of post intervention data collection for a duration of 6 months till October 2016 to study the incidence of CLABSI and hand hygiene compliance among the nurses. Another objective assessment test was done at the end of 6 months to assess the quality improvement in central venous catheter care and the effectiveness of our educational program. An infection control nurse reported the percentage of compliance with regard to hand hygiene during the central venous catheter care in the ICU patients before and after the workshop. This nurse is part of the infection control team of the hospital who visits ICU daily. The others nurses were not aware when the assessment was being carried. The same nurse did both pre and post intervention assessment.

Statistical analysis was done using the SPSS Software version 20. Qualitative variables are compared using Mann Whitneys U test and categorical data were analysed using chi square test or fishers exact test where ever feasible.

**Table 1:** Central line associated blood stream infection data

	*Pre-intervention*	*Post-intervention*
Number of patients	276	352
Catheter day	1200	1870
CLABSI rate	12.5	8.6
CLABSI case	15	16

CLABSI: Central Line Associated Blood Stream Infection

**Table 2:** Data from the educational intervention

	*Pretest*	*Posttest*	*3 Months Posttest*	*6 Months Posttest*
Marks	15.9 ± 3.3	17.7 ± 2.1	17.3 ± 1.9	17.0 ± 2.0
Non-compliance with hand Hygiene (%)	53.4	17.1	33.7	51.7

Marks are in Mean ± Standard Deviation

## RESULTS

A total of 34 nurses were enrolled for the study. The pretest marks obtained by them was 15.9 +/− 3.35 (14.77 − 17.11). The marks secured in the objective assessment test immediate post intervention, post 3 months and post 6 months were 17.76 +/− 2.1 (17.03 − 18.49), 17.35 +/− 1.9 (16.6 −1 8.03) and 17.00 +/− 2 (16.2 − 17.1). A statistically significant improvement in marks was only between the pretest and immediate post intervention test (*p*=0.011) with no significant rise during the subsequent 3 months and 6 months period.

The total number of catheter days were 1200 with a CLABSI rate of 12.5 per 1000 catheter days. There was a significant reduction (*P*=0.02) in CLABSI rates i.e. 8.6 post workshop with a total catheter day 1870 (Table 1).

The total number of opportunities, as reported by the infection control nurse, where hand hygiene steps could be adhered to, were 88 before the intervention, 53.4% of which were missed. Three months post test, the percentage of the non-compliance with hand hygiene was 33.75%, the reduction being statistically significant (*p*=0.02). 6 months post intervention, the percentage of the noncompliance with hand hygiene was raised to 51.75% (*p*=0.01) (Table 2).

## DISCUSSION

Our study an education based program on hand hygiene with a before after evaluation to assess the effect of the educational intervention on CLABSI rates showed that education reduced the rates of infection and also improved the hand hygiene compliance among nurses.

Central line associated bloodstream infections, are a major preventable cause of in hospital morbidity and mortality, the incidence being much more in the limited resource nations as compared to developed countries. The data on the CLABSI rates in India is variable; Patil et al and Kaur et al reported CLABSI rates of 18.5% and 2.8 per 1000 catheter days respectively.^9,10^ Mehta et al reported a CLABSI rate 7.9 per 1000 catheter days in 12 ICUs of seven hospitals.^11^ In our study, the CLABSI rates in the 6 months pre intervention period was 12.5 per 1000 catheter days which was far in excess of than the national average. In view of the high CLABSI rates, hand hygiene and vigilant nursing care were considered the most basic steps that needed reappraisal.

Hand hygiene is an integral part of any infection control program.^12,13^ Healthcare workers get contaminated by organisms from colonised patients, inter-personnel contacts and the immediate environment posing a threat of infection transmission. As per CDC recommendations, hand hygiene should be performed before donning gloves, before and after palpating the site of catheter insertion, before and after inserting the catheter, before and after accessing, replacing, repairing or dressing the catheter and after removal of gloves.^14^ The World Health Organisation leads a global effort with 170 countries signed up to ‘Clean Care is Safer Care Campaign’ to promote hand hygiene compliance. However, the best practice for hand hygiene is still unestablished.^15^ Hand washing and hand hygiene compliance was the main focus of the educational program in this study. It consisted of a didactic lecture and a bedside demonstration on hand hygiene practice.

The objective assessment tests were done to analyse the knowledge among the ICU nurses. The marks obtained escalated in significant proportions in the immediate post intervention period but there was no major change in the post intervention 3 months and 6 months duration. This emphasises the need of educational programs, effective communications, feedbacks on the infection rates and strict rules to motivate hand hygiene compliance among the healthcare workers. Chavali et al.^15^, Harris et al.^16^ and Mathai et al.^17^ asserted the use of multimodal techniques to improve performance and hand hygiene compliance among healthcare workers.

Hand hygiene compliance is best measured by direct or remote behavioural observation.^18^ In our study, the infection control nurse observed the total number of opportunities and the number of missed opportunities for hand hygiene compliance both before and after the educational program. The ICU nurses were unaware that they were being observed. The percentage of missed opportunities had a drastic reduction in the immediate post intervention 3 months duration but again increased in the 6 months post intervention period. Though this may indicate the need for retraining and repeat reminders to the nurses as you have stated, the good compliance during the study period may be due to a “Hawthorne effect” and the compliance came back to baseline once the study was over.

Implementation of education programs among the hospital staff using evidence based guidelines has a beneficial impact on the reduction of CLABSI.^20^ The incidence of CLABSI was reduced from 3.29 to 2.36 per 1000 catheter days after a single day education program on in service physicians. ^21^ A before after evaluation on 241 patients revealed that training healthcare workers could decrease the rate of catheter associated infections by 41.7%.^22^ Guembe et al reported insufficient impact of educational interventions in decreasing catheter associated infections.^22^ The studies, however, varied with regard to the type of intervention, the target population, time of study, prevalent infection rates and the data analysis methods.^24^ Our study was an education based program on hand hygiene, comparing the CLABSI rates 6 months before and 6 months after the intervention. Nurses, being the frontline of CLABSI control and prevention, the program was designed for the nurses.^24^ CLABSI rate was reduced by 31.2% in the postintervention period. Though it had a substantial impact on infection control, an ideal infection rate was not achieved which may be due to the initial high rate of infections, lack of knowledge on other prevention strategies and partially due to noncompliance with hand hygiene practices even after the workshop (missed opportunities). Jaggi et al after implementing the six components of the INICC approach simultaneously found significant reduction in the CLABSI rate in India, which remained stable during 36 months of follow-up. This Multi-centric trial demonstrated a reduction in CLABSI rates drom 6.4 to 3.9 per 1000 catheter days.^25^

Most of the previous studies on the reduction of CLABSI rates were based on bundled interventions and the effect could not be attributed to any single step.^26^ The evidence on all individual components of the care bundle were lacking and increased the cost of treatment. The duration of intervention was not clear in most of the studies and only few of them provided data on the maintenance of intervention effects. A majority of the studies were observational and failed to assess reliability of the hand hygiene observations. They provided insufficient description of the educational interventions and hence could not be replicated. Our study focused on the single, simple and cost effective component of hand hygiene. The data collection was both observational as well as the impact of intervention in reduction of CLABSI rates was assessed. Moreover, the steps and practice of hand hygiene are easy to demonstrate and replicate.

## LIMITATIONS

Inspite of the significant reduction in CLABSI rates, there were certain limitations in this study. Firstly, the bundle interventions for catheter care were not considered. Secondly, the impact on in hospital morbidity, mortality and impact on length of stay and ICU costs were not assessed. Thirdly, the sites of catheter insertion, severity of illness, presence of other catheters and the organisms causing the infections were not analysed. In addition, this was an uncontrolled study performed over a long duration and hence cannot exclude other contributing factors that could possibly reduce the infection rates. Fourthly, the education programme also included education on “Basic clinical practices for reduction of central venous catheter infections”, hence we cannot say with certainty, that the reduction in CLABSI rates is only due to an improvement in hand hygiene practices. It may well be a combination of both.

## CONCLUSION

Our study showed that CLABSI rates can be reduced by improving compliance with the basic steps of hand hygiene. Educating and implementing hand hygiene practice is easy but sustaining it over time is a challenge and requires regular training, motivation and reminders among the healthcare workers.
